# Editorial: Microbiology of radioactive environments

**DOI:** 10.3389/fmicb.2024.1352802

**Published:** 2024-01-29

**Authors:** Sudhir K. Shukla, Rok Tkavc, Haitham Sghaier

**Affiliations:** ^1^Biofouling and Biofilm Processes Section, Water and Steam Chemistry Division, Bhabha Atomic Research Centre Facilities, Kalpakkam, India; ^2^Homi Bhabha National Institute, Mumbai, India; ^3^Henry M. Jackson Foundation for the Advancement of Military Medicine, Bethesda, MD, United States; ^4^Department of Microbiology and Immunology, Uniformed Services University of the Health Sciences, Bethesda, MD, United States; ^5^Laboratory “Energy and Matter for Development of Nuclear Sciences” (LR16CNSTN02), National Center for Nuclear Sciences and Technology (CNSTN), Sidi Thabet Technopark, Ariana, Tunisia; ^6^Universite de la Manouba, ISBST, BVBGR-LR11ES31, Biotechpole Sidi Thabet, Ariana, Tunisia

**Keywords:** bioremediation, editorial, environments, microbiology, radioactive

Microbial life thrives in extreme and inhospitable environments, including radioactive environments. From nuclear power plants to radioactive waste sites, these environments pose unique challenges and opportunities for microbial life. This editorial aims to shed light on recent discoveries and implications of studying the microscopic inhabitants of these radioactive realms.

Radioresistance has been observed in a broad range of organisms, yet the exact evolutionary origins of this complex phenotype remain an unresolved query. Four possible theories for the appearance of radiation resistance have been proposed: (i) it is based on proteins that originated in ancestral hyperthermophiles (DiRuggiero et al., 1997; Di Giulio, 2003), (ii) it emerged incidentally following adaptation to dry conditions (Mattimore and Battista, 1996), (iii) it has an interplanetary origin (McKay et al., 1996), or (iv) it is a unique molecular reflection of the early Earth's resilience as a large set of data suggests that radiation was a driving force during chemical and early biochemical evolutions (Draganic and Adloff, 1993). For example, carbon coatings were recorded around uranium-bearing apatite grains in early Archean samples formed more than 3.85 billion years ago (Mojzsis et al., 1996). Also, for instance, given that manganese-rich niches with elevated natural ionizing-radiation (IR) levels constitute one of the most notable attributes of sediments located 20–100 meters below the seafloor, it is noteworthy that bacteria closely related to *Deinococcus geothermalis* have been successfully isolated from deep ocean subsurface environments (68–118 meters below the seafloor) (Kimura et al., 2003). Furthermore, it is important to note that after the major Proterozoic rise in oxygen 2.3 billion years ago (Holland, 2006), uranium, insoluble in water under anaerobic conditions, subsequently became readily soluble in water in the presence of oxygen. Thus, a consequence of the appearance of oxygen, in association with bacterial leaching abilities, was the natural advent of the Earth's nuclear reactors (Draganic and Adloff, 1993) containing a complex microbiota (Pedersen et al., 1996). It is calculated that about 100 million Oklo-type reactor sites could have been active in the past (Draganic and Adloff, 1993). A typical natural nuclear reactor with a core radius of two meters has a total dose rate of 47.4 Gy/h (Draganic and Adloff, 1993).

Radioactive waste is a persistent global concern, and finding efficient and cost-effective methods to neutralize or remove these contaminants is paramount. Microbes with relevant metabolic capabilities offer promising solutions to pertinent questions. In what ways can IR-resistant bacteria provide eco-friendly solutions for the bioremediation of uranium, and what factors determine their effectiveness? How does the utilization of innovative proteotyping approaches contribute to a deeper understanding of microbial communities thriving in “extreme” environments like a pool storing radioactive sources? How do microorganisms assume pivotal roles in deep geological repositories, potentially impacting the safety of storing radioactive waste? What can the exploration of IR-resistant prokaryotes' genomes reveal about their adaptive strategies in response to environmental challenges? What mechanisms underlie the impressive resilience of microbial communities in nuclear reactors when faced with long-term fluctuations in conditions? These and similar questions are addressed by the five articles featured in this Research Topic “Microbiology of Radioactive Environments”.

Martínez-Rodríguez et al. explored how the uranium (U) biomineralization capacity of *Microbacterium* sp. Be9, wherein U precipitates with ligands like phosphate (P), was influenced by the presence or absence of various P sources. The biotechnological potential of the Be9 strain for U bioremediation is highlighted in this research, showcasing its dual capability to both precipitate and solubilize the radionuclide under varying environmental conditions.

In the study of Pible et al., metaproteomic data were acquired and compared against a generalist database, revealing insights into the taxonomy (*Acidovorax, Caulobacter* and *Sphingomonas*) and metabolism of the microbial components in biofilms collected from a pool storing radioactive sources in a nuclear facility. This research introduced a novel approach for remotely sampling biofilms together with a pioneering technique to process MS/MS spectra data acquired post-protein extraction. Also, it demonstrated that metaproteomics can directly analyze biofilm microbial compositions with as little as 0.02 mg of material, offering a valuable tool for proteotyping.

The review of Ruiz-Fresneda et al. offers insights into how microorganisms near deep geological repositories (DGRs) can impact their safety, particularly through radionuclide-microbial interactions, potentially enhancing the effectiveness of planned radioactive waste repositories. The significance of this study lies in recognizing the presence of naturally occurring microorganisms within filling and sealing materials, like bentonite clay, which come into direct contact with the metal canisters containing radioactive waste. Bacterial communities in the bentonites, such as Opalinus, Boom and Spanish clay, and their potential activities, require consideration after DGR closure.

Shin et al. investigated the genome of *Deinococcus radiopugnans* DY59, revealing a total of 36 insertion sequence (IS) elements across six IS families, including five unclassified IS elements. The research focused on active transposition during short-term oxidative stress, induced by an 80 mM hydrogen peroxide (H_2_O_2_) treatment. ISDrpg2 and ISDrpg3 elements from the IS4 family transposed into a carotenoid synthesis gene (QR90_10400), causing a loss of pigmentation. DY59 displayed a high minimum inhibitory concentration (MIC) of 300 mM H_2_O_2_. Expression levels of catalase and LysR family regulators were assessed *via* real-time quantitative reverse transcription PCR (qRT-PCR), providing insights into this bacterium's response to oxidative stress.

Van Eesbeeck et al. focused on the microbial community in the BR2 nuclear research reactor in Mol, Belgium, which undergoes cyclic operational phases and shutdowns, leading to shifts in water basin conditions. Using 16S rRNA amplicon sequencing, the research found distinctive microbial profiles during cycles. Despite a 1-year gap between sampling campaigns, similar patterns emerged, indicating system stability over 2 years. The study correlated these microbial shifts with physico-chemical changes. Additionally, radiation was linked to decreased cell numbers, while temperature had the opposite effect. The microbes appeared to adapt by utilizing chemoautotrophy and recycling dead cells in this highly nutrient-scarce environment.

These studies provide intriguing case studies of microorganisms in radioactive environments, uncovering their remarkable adaptability and interactions with their environment. As we explore the microbiology of radioactive environments, we not only gain deeper insights into the remarkable resilience of life on Earth but also unlock valuable knowledge with potential applications in biotechnology, bioremediation and astrobiology. This frontier of microbial life reminds us of the remarkable adaptability and tenacity of life, urging us to explore and respect the complex interplay between microbiology and the radioactive world.

## Author contributions

SS: Writing – review & editing. RT: Writing – review & editing. HS: Writing – original draft, Writing – review & editing.

## References

[B1] Di Giulio M. (2003). The universal ancestor and the ancestor of bacteria were hyperthermophiles. J. Mol. Evol. 57, 721–730. 10.1007/s00239-003-2522-6 14745541

[B2] DiRuggiero J. Santangelo N. Nackerdien Z. Ravel J. Robb F. T. (1997). Repair of extensive ionizing-radiation DNA damage at 95 degrees C in the hyperthermophilic archaeon *Pyrococcus furiosus*. J. Bacteriol. 179, 4643–4645. 10.1128/jb.179.14.4643-4645.1997 9226280 PMC179306

[B3] Draganic I. G. Adloff J. P. (1993). Radiation, and Radioactivity on Earth and Beyond. London: CRC Press.

[B4] Holland H. D. (2006). The oxygenation of the atmosphere and oceans. Philosophical transactions of the royal society of London. Series B. Biol. Sci. 361, 903–915. 10.1098/rstb.2006.1838 PMC1578726 16754606

[B5] Kimura H. Asada R. Masta A. Naganuma T. (2003). Distribution of microorganisms in the subsurface of the manus basin hydrothermal vent field in Papua New Guinea. Appl. Environ. Microbiol. 69, 644–648. 10.1128/AEM.69.1.644-648.2003 12514053 PMC152445

[B6] Mattimore V. Battista J. R. (1996). Radioresistance of *Deinococcus radiodurans*: functions necessary to survive ionizing radiation are also necessary to survive prolonged desiccation. J. Bacteriol. 178, 633–637. 10.1128/jb.178.3.633-637.1996 8550493 PMC177705

[B7] McKay D. S. Gibson E. K. Thomas-Keprta K. L. Vali H. Romanek C. S. Clemett S. J.. (1996). Search for past life on Mars: possible relic biogenic activity in martian meteorite ALH84001. Science 273, 924–930. 10.1126/science.273.5277.924 8688069

[B8] Mojzsis S. J. Arrhenius G. McKeegan K. D. Harrison T. M. Nutman A. P. Friend C. R.. (1996). Evidence for life on earth before 3,800 million years ago. Nature 384, 55–59. 10.1038/384055a0 8900275

[B9] Pedersen K. Arlinger J. Hallbeck L. Pettersson C. (1996). Diversity and distribution of subterranean bacteria in groundwater at Oklo in Gabon, Africa, as determined by 16S rRNA gene sequencing. Mol. Ecol. 5, 427–436. 10.1111/j.1365-294X.1996.tb00332.x 8688960

